# Supplementary material for: Mesoscopic whole-brain T2*-weighted and associated quantitative MRI in healthy humans at 10.5 T
Source: bioRxiv. 2025 Apr 24:2025.04.21.649819. Preprint. [Version 1] doi: 10.1101/2025.04.21.649819 (PMC12190817; doi:10.1101/2025.04.21.649819)
Supplement: Supplement 1 [file media-1.docx]

**Supplemental materials for “Mesoscopic whole-brain T_2_^*^-weighted and associated quantitative MRI in healthy humans at 10.5 T”**

Jiaen Liu^1,2^, Peter van Gelderen^3^, Jacco A. de Zwart^3^, Jeff H. Duyn^3^, Yujia Huang^1^, Andrea Grant^4^, Edward Auerbach^4^, Matt Waks^4^, Russell Lagore^4^, Lance Delabarre^4^, Alireza Sadeghi Tarakameh^4^, Yigitcan Eryaman^4^, Gregor Adriany^4^, Kamil Ugurbil^4^ and Xiaoping Wu^4^

1 Advanced Imaging Research Center, UT Southwestern Medical Center, Dallas, TX, USA

2 Radiology, UT Southwestern Medical Center, Dallas, TX, USA

3 Advanced MRI section, NINDS, NIH, Bethesda, MD, USA

4 Center for Magnetic Resonance Research, Radiology, Medical School, University of Minnesota Twin Cities, Minneapolis, MN, USA

**Supplemental Figures**


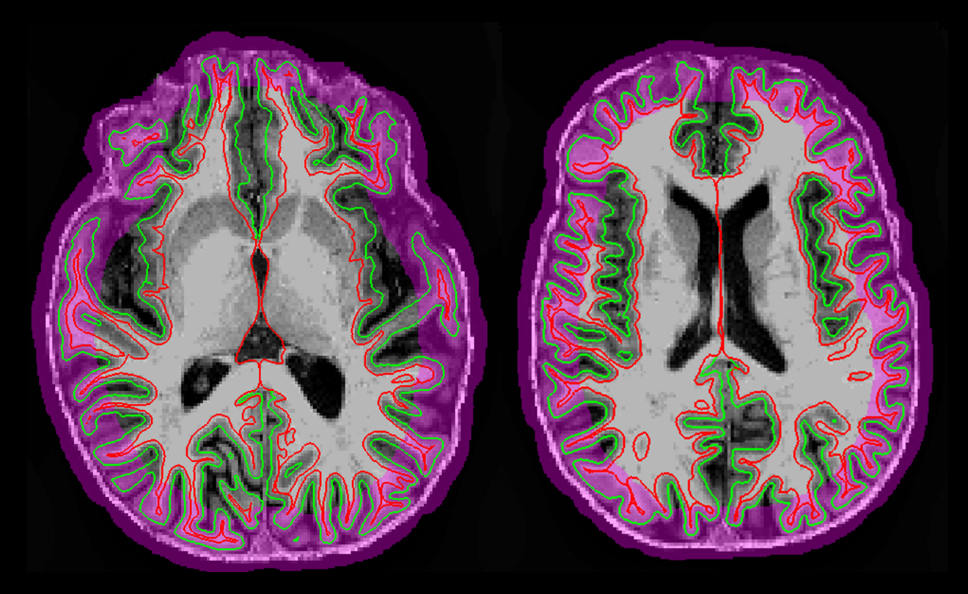


Fig. S1. Regions included for intrinsic SNR and *R*_2_^*^ contrast analysis near the periphery of the cerebrum as defined by the purple band. Two representative axial slices from one volunteer are shown as examples. Data analysis was performed within the purple band, along the selected cortical gray matter layer (as indicated by the green contour), and its adjacent white matter layer (as indicated by the red contour).


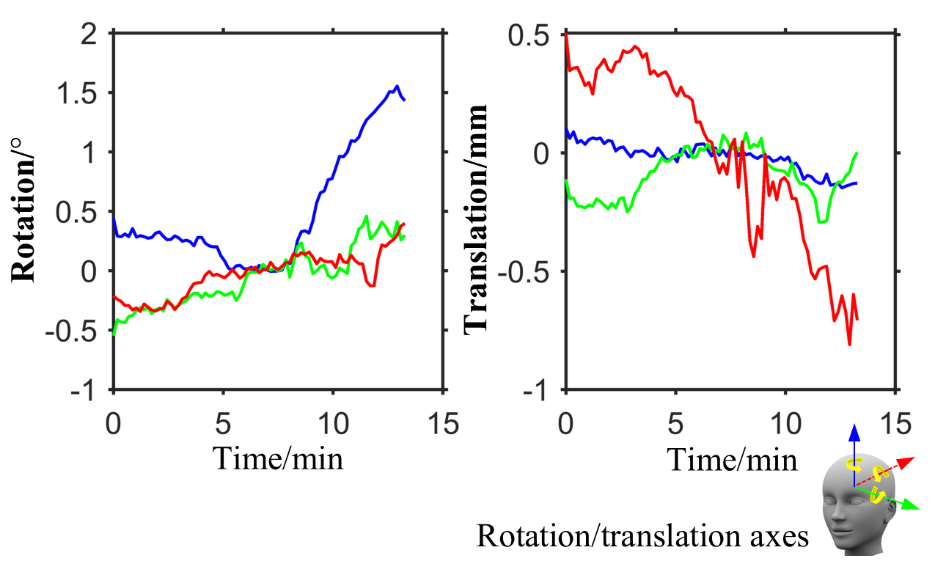


Fig. S2. Motion tracking based on navigators. Shown are the navigator-measured rotation and translation time courses in one volunteer during the scan. The three axes that define the rotation and translation are color coded as illustrated in the human head model on the bottom right. Note that moderate head motion was observed throughout the ~13.3 min 10.5 T scan associated with Fig. 1 in the main text.
